# Performance of nucleocapsid and spike-based SARS-CoV-2 serologic assays

**DOI:** 10.1371/journal.pone.0237828

**Published:** 2020-11-02

**Authors:** Zahra Rikhtegaran Tehrani, Saman Saadat, Ebtehal Saleh, Xin Ouyang, Niel Constantine, Anthony L. DeVico, Anthony D. Harris, George K. Lewis, Shyam Kottilil, Mohammad M. Sajadi

**Affiliations:** 1 Division of Clinical Care and Research, Institute of Human Virology, University of Maryland, Baltimore, Maryland, United States of America; 2 Division of Vaccine Research, Institute of Human Virology, University of Maryland School of Medicine, Baltimore, Maryland, United States of America; 3 Division of Epidemiology & Prevention, Institute of Human Virology, University of Maryland School of Medicine, Baltimore, Maryland, United States of America; 4 Department of Epidemiology and Public Health, University of Maryland School of Medicine Baltimore, Maryland, United States of America; 5 Department of Medicine, Baltimore VA Medical Center, Baltimore, Maryland, United States of America; Qatar University, QATAR

## Abstract

There is an urgent need for an accurate antibody test for severe acute respiratory syndrome coronavirus 2 (SARS-CoV-2). We have developed 3 ELISA methods, trimer spike IgA, trimer spike IgG, and nucleocapsid IgG, for detecting anti-SARS-CoV-2 antibodies. We evaluated their performance along with four commercial ELISAs, EDI™ Novel Coronavirus COVID-19 ELISA IgG and IgM, Euroimmun Anti-SARS-CoV-2 ELISA IgG and IgA, and one lateral flow assay, DPP® COVID-19 IgM/IgG System (Chembio). Both sensitivity and specificity were evaluated and the probable causes of false-positive reactions were determined. The assays were evaluated using 300 pre-epidemic samples and 100 PCR-confirmed COVID-19 samples. The sensitivities and specificities of the assays were as follows: 90%/100% (in-house trimer spike IgA), 90%/99.3% (in-house trimer spike IgG), 89%/98.3% (in-house nucleocapsid IgG), 73.7%/100% (EDI nucleocapsid IgM), 84.5%/95.1% (EDI nucleocapsid IgG), 95%/93.7% (Euroimmun S1 IgA), 82.8%/99.7% (Euroimmun S1 IgG), 82.0%/91.7% (Chembio nucleocapsid IgM), 92%/93.3% (Chembio nucleocapsid IgG). The presumed causes of false positive results from pre-epidemic samples in commercial and in-house assays were mixed. In some cases, assays lacked reproducibility. In other cases, reactivity was abrogated by competitive inhibition (spiking the sample with the same antigen that was used for coating ELISAs prior to performing the assay), suggesting positive reaction could be attributed to the presence of antibodies against these antigens. In other cases, reactivity was consistently detected but not abrogated by the spiking, suggesting positive reaction was not attributed to the presence of antibodies against these antigens. Overall, there was wide variability in assay performance using our samples, with in-house tests exhibiting the highest combined sensitivity and specificity. The causes of “false positivity” in pre-epidemic samples may be due to plasma antibodies apparently reacting with the corresponding antigen, or spurious reactivity may be directed against non-specific components in the assay system. Identification of these targets will be essential to improving assay performance.

## Introduction

There is an urgent need for an accurate serologic test for severe acute respiratory syndrome coronavirus 2 (SARS-CoV-2). Antigen and antibody detection methods play important roles in disease management and control of COVID-19. Due to the high specificity of the reverse transcriptase Real Time PCR (rRT-PCR) for detecting the presence of the virus during the acute phase [1], it is considered the gold standard for COVID-19 clinical testing [2]. Antibody testing currently plays a limited role in testing patients [3], due to the potentially long window period of 1 to 2 weeks after onset of symptoms [4,5]; however, they can be critical in other aspects of the disease. Antibody based tests can be used for detection of previously infected patients for population-level surveillance, as a confirmatory assay for PCR testing, as a cost-effective method for primary diagnostic test in low-income settings, defining the antibody titers following vaccination, screening eligible convalescent plasma donors, and potentially determining protection against re-infection [6]. Among the four major structural proteins encoded by SARS-CoV-2 genome, i.e spike (S), envelope (E), membrane (M), and nucleocapsid (N) [7], the two latter proteins are highly immunogenic and therefore are widely used in serologic assays [8].

At the time of writing this paper, the U.S. Food and Drug Administration (FDA) has authorized 51 antibody tests under Emergency Use Authorization (EUA) [9]. However, because of the long time to seroconversion, as well as lack of specificity, the CDC currently only recommends serologic testing in certain clinical situations, such as presentation after 9 days of illness onset, and those presenting with late complications of COVID-19 [3].

Despite their importance in disease management, the performance of many commercially available SARS-CoV-2 serologic tests have not been fully evaluated with large panels of samples, thus, their utility is questionable [3]. A number of these tests have been recalled due to poor performance.

Because seasonal coronaviruses have conceivably infected the majority of the human population, cross reactivity to the common coronaviruses is an important concern in developing SARS-CoV- 2 serology tests. These tests include ELISA, and chemiluminescent microparticle immunoassay (CMIA) or lateral flow immunoassays (as point of care tests), which target specific antibodies against viral spike or nucleocapsid proteins. We have developed 3 ELISA tests for detecting anti-SARS-CoV-2 antibodies and evaluated their performance characteristics along with to four commercial ELISA and one lateral flow assays.

## Materials and methods

### Patient samples

A total of 100 plasma samples collected from PCR-confirmed COVID-19 patients, age between 27–89 (40% female) were used as the positive group. The samples were from patients hospitalized at the University of Maryland Medical Center with a diagnosis of COVID-19 in April-May 2020. Where possible, the last available sampling time point was used (0–51 days post symptom onset; median 12 days). In addition, a total of 300 pre-epidemic plasma/sera samples that were collected before the COVID-19 epidemic (2005–2019) served as negative controls.

Development and optimization of the in house ELISA methods was initially performed using eight PCR-confirmed COVID-19 and eight pre-epidemic samples. The performance of the assays were then evaluated using all 100 positive and 300 negative samples.

A summary of the samples used in this study are noted in Table 1. Samples were obtained from protocols approved by the University of Maryland, Baltimore IRB (written informed consent obtained for pre-epidemic samples, and waiver of consent obtained for COVID-19 patient samples).

**Table 1:** Patient samples used in this study.

Positive Samples	COVID-19, PCR confirmed	100
Pre-epidemic samples (2005–2019)	HIV infected	66
	Healthy control (HIV vaccinated)*	70
	Healthy controls (Blood donors)	164
Total		400

*Part of IHV01 vaccine study: NCT03505060.

### In-house ELISAs

We developed three different ELISAs for the qualitative evaluation of the human serum or plasma for detection of IgG or IgA antibodies in human serum or plasma, targeting the SARS- CoV-2 trimer spike protein (IgG and IgA ELISA) and IgG antibodies targeting the SARS-CoV-2 nucleocapsid antigen (IgG ELISA). For the development and optimization of the assays, eight true positive and eight true negative samples were used.

#### A. Recombinant proteins

SARS-CoV-2 trimer spike prefusion and trimerization-stabilized ectodomain (LakePharma, Hopkinton, MA, Cat. #46328) were used for developing the trimer spike IgG and IgA ELISAs. For the nucleocapsid IgG ELISA, the SARS-CoV-2 nucleocapsid protein was produced. Briefly, the nucleic acid sequence of the SARS-CoV-2 nucleocapsid (residues 1–419; isolate Wuhan-Hu- 1; GenBank MN908947.3) was synthesized and cloned into OriGene expression vector by BlueHeron (Bothell, WA) along with C-terminal His tag. The plasmid was expressed in FreeStyle 293-F cells (Thermo Fisher Scientific, Waltham, MA; Cat# R79007) and the transfected cells were harvested on day 7 post transfection. The recombinant nucleocapsid was affinity purified from the cell lysate by Ni-NTA Agarose (Qiagen, Cat# 30210) under native conditions. The purity of the purified nucleocapsid was evaluated by SDS- PAGE. (S1 Fig).

#### B. Developing and performing the in house ELISAs

The in-house ELISAs were performed by coating Immulon 2 HB 96-well flat bottom plates (Immuno Chemistry Technologies, Bloomington, MN) with 0.1 μg/well of the trimer spike or nucleocapsid proteins in TBS overnight at 4°C. Following blocking with Blotto (10% dried milk in TBS and 0.1% NP-40), the 1:100 diluted samples in Blotto was added and incubated for 1 hour at 37°C. After washing the plates with TBS, the antigen specific antibodies were incubated with alkaline phosphatase labeled goat anti human IgG or IgA antibodies (Southern Biotech, Birmingham, AL) for 1 hour at 37°C. After washing the wells, the BluePhos® Microwell (Seracare, Milford, MA) was added to each well as Substrate for 15 min at 37°C. The enzymatic reaction was stopped by adding KPL APstop™ Solution (Seracare, Milford, MA) and the signal was read at 650 nm.

### Commercial assays

#### A. ELISAs

**The EDI™ Novel Coronavirus COVID-19 ELISA IgG** (Epitope Diagnostics, Inc. San Diego, CA, Cat. # 1032) was performed according to the manufacturer’s instruction. The assay is an ELISA that detects specific IgG antibodies in human serum or plasma by binding SARS-CoV-2 nucleocapsid on the plates. The cut-off value for negative and positive results was calculated by adding the calculated average of negative controls to 0.18 and multiplying to 0.9 and 1.1, respectively.

**The EDI™ Novel Coronavirus COVID-19 ELISA IgM** (Epitope Diagnostics, Inc. San Diego, CA, Cat. #1033) was performed according to the manufacturer’s instruction. The assay is based on the capture of total IgM antibodies in human serum or plasma and then detects antibodies binding SARS-CoV-2 nucleocapsid. The cut-off value for negative and positive results was calculated by adding the calculated average of negative controls to 0.1 and multiplying by 0.9 and 1.1, respectively.

**Euroimmun Anti-SARS-CoV-2 ELISA IgG and IgA** (Euroimmune, Germany; Cat # EI 2606–9601 G and EI 2606–9601 A, respectively) were performed according to the manufacturer’s instructions. The Euroimmun Anti-SARS-CoV-2 IgG and IgA tests are separate ELISAs that detect antibodies against the S1 subunit of the SARS-CoV-2 spike protein. Results were determined as a ratio of the signal of the samples to the average signal of calibrators. The interpretation of the calculated ratios was performed as manufacturer’s recommendation. A ratio < 0.8 was considered as not elevated (Negative), ≥0.8 to <1.1 as indeterminate (borderline) and ≥1.1 as elevated (Positive).

#### B. Lateral flow assay

The DPP® COVID-19 IgM/IgG System (Chembio Diagnostics Systems Inc. Medford, NY) is a Dual Path Platform (DPP) system for qualitative rapid detection and differentiation of IgM and IgG antibodies to SARS-CoV-2 nucleoprotein in capillary “fingerstick” whole blood, venous whole blood, serum, and plasma samples. The test was performed at room temperature according to the manufacturer’s instruction. The results were read using the DPP Micro Reader that provides an objective result (positive and negative).

### Data analysis

#### A) Cutoff calculation for in-house ELISAs

The cutoff values for the in-house ELISAs were determined by two different approaches. In the first approach, the first cutoff value was determined using the average OD plus 3 standard deviation (SD) of all 300 pre-epidemic samples. In the second approach, to determine the cutoff value a ROC curve was generated for each assay, using SPSS Statistics for Windows, version 16.0 (SPSS Inc., Chicago, Ill., USA). The area under curve (AUC) and the sensitivity and specificity at all the cutoff points were also calculated. Then, the optimal cutoff point was selected based on the point with the highest Youden index J (J = sensitivity + specificity—1). This is the point on the curve in which the distance to diagonal line (line of equality) is maximum [10].

For each approach, the samples with ODs greater than the determined cutoff values were considered as positive and the samples with ODs equal or less than the cutoff values were considered as negative. The optimal cutoff value for each assay was determined based on the cutoff with the highest accuracy, which defined as the percentage of true positive and negative results divided to the total number of the samples [11]. For calculation of the sensitivity and specificity of the commercial ELISAs the borderline results were excluded from calculation.

#### B) Calculation of the performance characteristics of all assays

Sensitivity and specificity of the assays were defined as their true positive and true negative rates, respectively. The positive status of the positive group was confirmed by rRT-PCR, and the negative status of the negative group was based on the time of sampling which was from pre-epidemic period. To determine the performance of the tests, the AUC (for ELISA Tests) and accuracy (for all assays) were calculated. In our study, all of the pre-epidemic samples are considered clinically negative and all of the PCR confirmed samples considered clinically positive, thus the accuracy is defined by the patient’s clinical status.

### Soluble antigen competition experiments to determine specificity of presumptive false positive reactions

Antigen specificity of presumptive false positive reactions was assessed by using soluble SARS- CoV-2 trimer spike (LakePharma, Worcester, MA; Cat. #46328) or nucleocapsid (home-made protein mentioned above) antigens to determine whether they inhibit reactivity of the specimen with the corresponding plate-bound antigen. For each assay the dilution of the starting plasma and concentration of the antigens used for the competition assay necessary to convert a true positive sample to negative (or at least a 3-fold reduction) were determined. Depending on the assay, concentration of antigen used were 100–500 μg/ml. Soluble trimer spike or nucleocapsid was pre-incubated with presumable false positive samples (direct plasma for spike and 1:100 plasma for nucleocapsid) for two hours at room temperature with shaking (Fisher Scientific isotemp, 125 RPM). To compensate for dilution of the specimens by pre-incubation, each sample also pre-incubated with 100 μg of bovine serum albumin (BSA) in PBS as control. After pre-incubation, the plates were evaluated by the corresponding assays as described above. To check the effect of spiking, each experiment were performed with negative and positive samples (with low or intermediate ODs) as control.

## Results

### Determination of the optimal cutoff for in-house ELISAs

The cutoff values were calculated based on two different approaches and the optimal cutoff for each assay was selected based on the cutoff value with the higher accuracy of each assay. Table 2 shows the performance of the in-house ELISAs. In the trimer spike IgG and IgA ELISA tests, the cutoff values calculated by ROC curve provided higher accuracies; thus, these values were selected for further evaluation of the assays. For the nucleocapsid ELISA, the calculated cutoff values with the two approaches was partially equal (0.19 and 0.20).

**Table 2:** Performance of the in-house ELISAs using different calculated cutoff values.

Method		Trimer Spike IgA	Trimer Spike IgG	Nucleocapsid IgG
Mean Neg. OD + 3 SD	Cutoff	0.12	0.19	0.20
	True Positive	91/100	90/100	89/100
	True Negative	295/300	296/300	295/300
	Indeterminate	N/A	N/A	N/A
	Sensitivity (95% CI)	91.0% (85.4–96.6)	90.0% (84.1–95.9)	89.0% (82.9–95.1)
	Specificity (95% CI)	98.3% (96.8–99.8)	98.7% (97.4–100)	98.3% (96.8–99.8)
	Accuracy (95% CI)	96.5% (94.7–98.3)	96.5% (94.7–98.3)	96.0% (94.1–97.9)
ROC Curve	Cutoff	0.15	0.28	0.19
	True Positive	90/100	90/100	89/100
	True Negative	300/300	298/300	295/300
	Indeterminate	N/A	N/A	N/A
	Sensitivity (95% CI)	90.0% (84.1–95.9)	90.0% (84.1–95.9)	89.0% (82.9–95.1)
	Specificity (95% CI)	100% (98.7–100)	99.3% (98.4–100)	98.3% (97.8–99.8)
	Maximum J index	0.900	0.893	0.873
	Accuracy (95% CI)	97.5% (96.0–99.0)	97.0% (95.3–98.7)	96.0% (94.1–97.9)
	AUC	0.975	0.966	0.976

### Evaluation of the performance of all immunoassays

**Fig 1:**
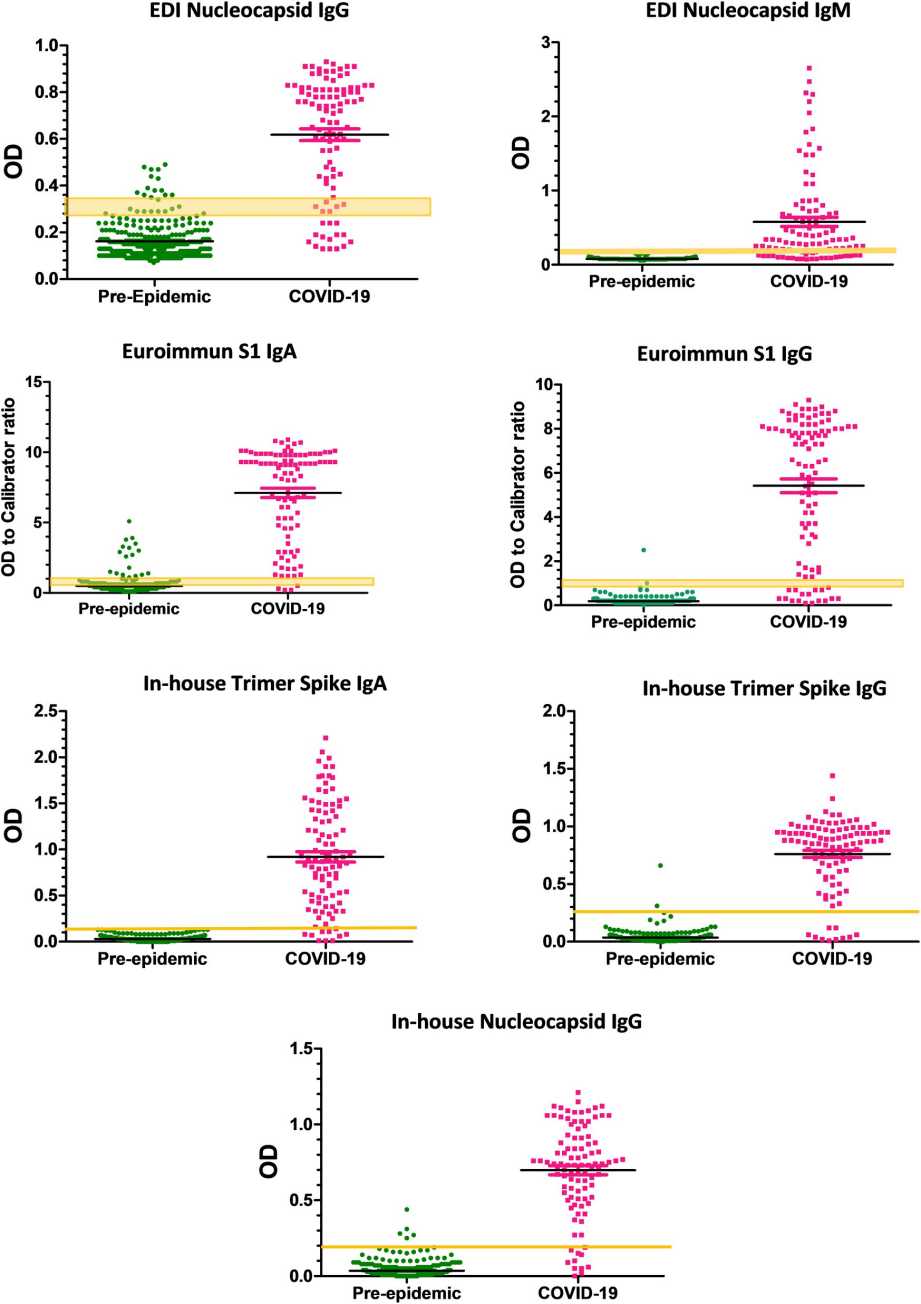
SARS-CoV-2 antibody assays data distribution. The data obtained for pre-epidemic and COVID-19 samples with all evaluated assays. The yellow lines show the cut-off values or ranges recommended by commercial assays or calculated cutoff values for in-house ELISAs. Black lines indicate median values with interquartile ranges.

To determine the performance of the assays, the ROC curve analysis was performed for all ELISA assays. The obtained data are shown in Table 3 and Fig 1, and the supplementary file, S1 Appendix, shows individual data from all experiments.

**Table 3:** Performance of commercial assays.

	EDI IgG	EDI IgM	Euro IgA	Euroimmun IgG	ChemBio IgM	ChemBio IgG
True Positive	82/97	70/95	95/100	82/99	82/100	92/100
True Negative	274/288	299/299	266/284	297/298	275/300	280/300
Indeterminate	15/400	6/400	16/400	3/400	N/A	N/A
Sensitivity (95% CI)	84.5% (77.3–91.7)	73.7% (64.8–82.6)	95.0% (90.7–99.3)	82.8% (75.4–90.2)	82.0% (74.5–89.5)	92.0% (86.7–97.3)
Specificity (95% CI)	95.1% (92.6–97.6)	100% (98.7–100)	93.7% (90.9–96.5)	99.7% (99.1–100.0)	91.7% (88.6–94.8)	93.3% (90.5–96.1)
Accuracy (95% CI)	92.5% (89.9–95.1)	93.7% (91.3–96.1)	94.0% (91.6–96.4)	95.5% (93.5–97.5)	89.3% (86.3–92.3)	93.0% (90.5–95.5)
AUC	0.944	0.964	0.970	0.966	NA	NA

### Soluble antigen competition experiments

The in-house trimer spike IgA ELISA had no false positives and was not evaluated by soluble antigen competition. For the in-house trimer spike IgG ELISA, the one sample that was repeatedly false positive turned negative with spiking. For the in-house nucleocapsid IgG ELISA, four samples that were repeatedly false positive samples turned negative when nucleocapsid was added. The Eurommun SARS-CoV-2 IgG had one false positive that remained positive after spiking. Of the 18 false positive results of Euroimmun SARS-CoV-2 IgA, only one sample decreased substantially and turned to negative after adding the trimer spike protein. For the EDI SARS-CoV-2 IgG, 8 of 22 false positive and/or indeterminate pre-epidemic samples changed to negative when treated with the nucleocapsid protein. The false positive samples in the DPP rapid assay were rerun a number of times, with varying results obtained each time (almost half of the samples turned from positive to negative, or negative to positive on retesting). As such, they were excluded from further analysis in the spiking experiments. A summary of the spiking experiments can be found in Table 4 and the supplementary file, S1 Appendix, has details of each experiment.

**Table 4:** Summary of the soluble antigen competition experiments.

Assay	Sample Status	Number of samples	Number (%) of samples that turned negative
In-house Trimer Spike IgG	False positive	4*	4 (100%)
	Indeterminate	NA	-
In-house Nucleocapsid IgG	False positive	1*	1 (100%)
	Indeterminate	NA	-
EDI IgG	False positive	14	4 (29%)
	Indeterminate	8ǂ	4 (50%)
Euroimmun IgA	False positive	18	2 (11%)
	Indeterminate	16	1 (6%)
	False positive	1	0 (0%)
Euroimmun IgG	Indeterminate	2	1 (50%)

* One sample turned negative on retesting and not included.

ǂ 8 out of 12 indeterminate pre-epidemic samples were tested in the Soluble Antigen Competition Experiments NA = Not Applicable.

## Discussion

Despite an urgent need and extensive efforts, the development of an efficient and fully validated serologic assay for detecting specific anti-SARS-CoV-2 antibodies are not yet available. In the present study, we developed and evaluated three ELISAs for detecting anti-trimer spike antibodies (IgG and IgA) and anti-nucleocapsid antibodies (IgG), and determined their performance along with 5 commercial immunoassays, including 4 ELISAs (EDI™ Novel Coronavirus COVID-19 IgG and IgM, Euroimmun SARS-CoV-2 IgG and IgA) and one lateral flow immunoassay (DPP® COVID-19 IgM/IgG). For evaluation of the assays, we used 100 COVID- 19 PCR-confirmed samples from hospitalized patient and 300 pre-epidemic samples taken before the emergence of the disease in December 2019. The ratio of pre-epidemic to post-epidemic samples was 3:1 as we were particularly interested in studying the specificity of the assays and reasons for false positivity.

SARS-CoV-2 has four structural proteins [12–14], and among them the spike and the nucleocapsid are considered the main immunogens and are widely used in immunoassays [8]. It has been shown that the anti-nucleocapsid antibodies appear earlier than the spike antibodies [15]. Therefore, applying the anti-nucleocapsid antibodies in ELISA tests may increase the clinical sensitivity of the assay, if samples are drawn early. The nucleocapsid is a protein with a small size that can easily be produced and purified in prokaryotic or eukaryotic hosts in vast quantities. Homology analysis show that the SARS-CoV-2 nucleocapsid has 90% amino acid identity to MERS-CoV and 28 to 49 percent identity to other alpha and beta coronaviruses [16]. The same degree of similarity to other coronaviruses has also been seen in the spike protein, particularly the S2 domain [16]. It has been suggested that using the S1 protein (and particularly the receptor binding domain) will improve the specificity of the immunoassays [17].

One area of concern is the potential for cross-reactivity with the four human seasonal coronaviruses. This concern can be addressed by designing a chimeric protein containing specific immunogenic regions of the spike and/or the nucleocapsid protein. However, it seems that until such a protein becomes available, it is still possible to provide high-efficiency immunoassays using these spike and nucleocapsid antigens. Here, we optimized the assays with the minimum amount of coated antigen and conjugated detection antibodies. This design along with the precise determination of accurate cutoff points may potentially improve the sensitivity and specificity of the in-house ELISAs.

Of the assays that employed spike protein (trimer for the in-house or subunit S1 for the Euroimmun), those with the best specificities include the in-house trimer spike IgA (specificity 100%), in house trimer spike IgG (99.3%), and Euroimmun IgG (99.7%), although the Euroimmun test also had close to an additional 1% indeterminate rate. The Euroimmun IgA performed poorly with a 93.7% specificity and 4% indeterminate results. The higher specificity of the Euroimmun IgG in comparison to the IgA test has been demonstrated in other studies [18,19]. Of the assays that employed nucleocapsid, those with the best specificities include the EDI- IgM (100% specificity) and the in-house nucleocapsid IgG (specificity 98.3%).

Some studies have shown different times to seroconversion to SARS-CoV-2 depending on the severity of the disease [20,21], while those who are asymptomatic or minimally symptomatic may have lower or undetectable antibody levels [21,22]. Thus, both the timing of blood collections and severity of disease can potentially affect the sensitivity of the assays. Therefore the antibody based diagnostics assays are not appropriate methods for diagnostic purposes in the early phase of infection, though they have a role after 9 days of infection [3]. We had three PCR positive samples, collected within the first week after the symptoms, which did not seroconvert during the time point of our study by any of the assays. It is possible that with longer follow-up time, some of these patients would have seroconverted. Use of a different patient population (truly convalescent samples) would likely increase the sensitivity in all the assays tested. Thus, the observed test indices must be viewed based on our population characteristics.

The accuracy is a useful parameter for evaluation of the performance characteristics of different assays. In the evaluated assays, the in-house ELISAs show higher accuracy than the commercial ones. The accuracy of the in-house trimer spike-IgA, trimer spike-IgG and nucleocapsid-IgG ELISAs was 97.5, 97.0 and 96.0%, respectively. The highest accuracy in the evaluated commercial assays belonged to the Euroimmun-IgG with 95.5% and the lowest one belonged to the Chembio-IgM with 89.3% accuracy. It should be noted that, during the preparation of this paper, the FDA revoked the EUA for Chembio antibody test over concerns about accuracy of the test and high rate of false results [23]. This suggests that the test was not verified to perform as expected.

Although it is expected that the presence of IgM antibody proves an earlier diagnosis for most infectious diseases, our findings suggest that the IgM antibodies are not detected substantially earlier than IgG antibodies. In our study, the two evaluated IgM assays, EDI-IgM and Chembio- IgM, showed the lowest sensitivity in comparison to the other assays. In addition, we did not have any IgM positive and IgG negative cases among the 100 COVID-19 samples, whereas the opposite was clearly noted (leading to a low sensitivity of the IgM assays). Similar findings have been shown in other SARS-CoV-2 and SARS-CoV-1 serodiagnostic assays [22,24]. However, we did not analyze serial samples, and thus no definite conclusions can be made as to the kinetics of the IgG and IgM responses.

In order to determine the probable cause for the false positivity in the various test formats, the false positive samples underwent competition experiments. In these experiments, the positive samples were pre-incubated with the analyte antigen (the soluble form of the corresponding plate-bound antigen), and thus, any significant cross-reactivity should result in a negative ELISA by competitive inhibition. For the in-house spike IgG and nucleocapsid IgG tests, all the samples that were repeatedly false positive became negative after the spiking, indicating that there was true cross-reactivity between the antibodies in the pre- epidemic samples and the SARS-CoV-2 spike or nucleocapsid antigens. Likewise, for the EDI nucleocapsid IgG, all the samples that were repeatedly false positive became negative after the spiking, indicating true cross-reactivity between the antibodies in the pre-epidemic samples and the SARS-CoV-2 spike and nucleocapsid antigens. This type of cross-reaction can be corrected by using the sample diluent buffers containing peptides of conserved nucleocapsid or spike (depending of the assay) of alpha and beta coronaviruses, or by designing a chimeric protein that contains specific sequences of antigens for coating.

By contrast, the competition experiment with the false positives from the Euroimmun SARS- CoV-2 IgG and Euroimmun SARS-CoV-2 IgA assays shows that most of these samples (18 of 19) are cross-reacting with something other than the coated S1 antigen used in the Euroimmun IgA. To reduce the number of false positive results in these assays, procedures such as re- optimization of the assay including revising the buffers, and increasing the purity of the coated antigen should be considered. Finally, about half the Chembio spiking experiments showed major problems with reproducibility, with the controls turning from positive to negative or vice- versa. Because of this, any attempt at interpretation was abandoned, and it should also be noted again that the FDA has revoked the EUA for this product.

For the studies addressing the clinical performance of diagnostic tests, the characteristics of the study population should be carefully considered. As mentioned above, the length of symptoms and the clinical severity of COVID-19 patients are factors that can affect the clinical sensitivity of the serologic assays. In addition, the specificity of the assays were evaluated using healthy controls (and not those with known seasonal coronavirus infection), which can be affected the observed clinical specificity.

In conclusion, we have evaluated the performance of several in-house ELISAs that incorporate the SARS-CoV-2 trimer spike and nucleocapsid antigens and compared them to the performance of the commercially available tests. All three in-house ELISA tests performed well, with high sensitivity and specificity, without the use of indeterminate results. Although the specificity of the best of these assays is high, even a specificity of 99% can be problematic in areas of low prevalence of SARS-CoV-2 infection i.e, positive predictive value. A high specificity of these assays is paramount because false positive results may lead to inaccurate seroconversion status (important for diagnosis in special cases, prevalence data, and potentially protective immunity after vaccination and disease). It is essential to determine the cause of false positive results so that serologic assays can be improved. In addition, testing algorithms with more than one test may be necessary to rule out false positives by initial tests, such that has been the rule for HIV and hepatitis. Nevertheless, we have shown the potential of some assays to have high specificities, depending on their target isotypes, the antigens used, and establishment of cutoff values.

## Supporting information

S1 Fig SDS-PAGE analysis of the purified nucleocapsid protein. Lane 1. Protein Marker. Lane 2. The purified SARS-CoV-2 Nucleocapsid electrophoresed in the MOPS buffer shows the estimated molecular weight of around 47 KDa.
(TIF)

S1 Appendix Supplementary data. (XLSX)
