# Supplementary figures and images for: Performance of nucleocapsid and spike-based SARS-CoV-2 serologic assays
Source: PLoS One. 2020 Nov 2;15(11):e0237828. doi: 10.1371/journal.pone.0237828 (PMC7605638; doi:10.1371/journal.pone.0237828)

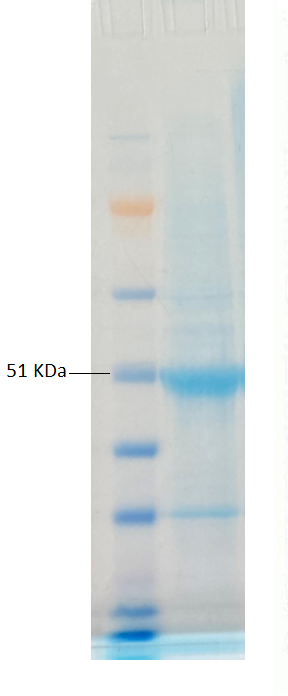

Supplement: S1 Fig — Lane 1. Protein Marker. Lane 2. The purified SARS-CoV-2 Nucleocapsid electrophoresed in the MOPS buffer shows the estimated molecular weight of around 47 KDa. (TIF) [file pone.0237828.s001.tif]
